# Strategies to promote oral health equity for immigrant populations in Canada and the USA: a scoping review protocol

**DOI:** 10.3389/froh.2026.1804288

**Published:** 2026-04-10

**Authors:** Rutik Ravindra Isai, Debora Lana Alves Monteiro, Liliani Aires Candido Vieira, Amrinderbir Singh, Jessi Robinson, Luiz Renato Paranhos, Yuri Wanderley Cavalcanti, Juliana Pereira da Silva Faquim

**Affiliations:** 1 School of Public Health, University of Saskatchewan, Saskatoon, SK, Canada; 2 Faculty of Dentistry, Federal University of Paraíba, João Pessoa, Brazil; 3 School of Dentistry, University of Louisville, Louisville, KY, United States; 4 College of Dentistry, University of Saskatchewan, Saskatoon, SK, Canada; 5 Health Sciences Library, University of Saskatchewan, Saskatoon, SK, Canada; 6 Faculty of Dentistry, Federal University of Uberlândia, Uberlândia, Brazil

**Keywords:** dental care, health equity, immigrants, oral health, refugees

## Abstract

**Introduction:**

Oral health inequities among immigrants in Canada and the United States remain a major challenge due to systemic, cultural, and socioeconomic barriers that limit access to dental services. Despite existing community and policy initiatives, there is no comprehensive synthesis of evidence addressing barriers, facilitators, and interventions aimed at promoting oral health equity for these populations.

**Objective:**

The purpose of this scoping review is to identify and map barriers, facilitators, and interventions related to promoting oral health equity among immigrants, refugees, asylum seekers, and non-permanent residents in Canada and the United States.

**Inclusion criteria:**

The review will include studies involving immigrants, refugees, asylum seekers, and non-permanent residents living in Canada or the United States that examine barriers, facilitators, or interventions related to oral health equity. Peer-reviewed studies and relevant grey literature published in English will be considered without date restrictions. Studies focusing exclusively on non-migrant populations or not addressing oral health will be excluded.

**Methods:**

This protocol follows the Joanna Briggs Institute (JBI) methodology for scoping reviews and the PRISMA-ScR reporting guidelines and is registered on the Open Science Framework (OSF) https://doi.org/10.17605/OSF.IO/PDNEM Searches will be conducted in MEDLINE (PubMed), Scopus, Web of Science, EBSCO databases, Embase, and relevant grey literature sources. Two reviewers will independently screen studies and extract data. Quantitative data will be summarized descriptively, and qualitative findings will undergo thematic synthesis. Results will be presented in tables and narrative summaries. Ethical approval is not required as the review uses published literature. Findings will be disseminated through peer-reviewed publications and conference presentations to inform policy and future research.

## 1 Introduction

Oral health is a fundamental component of overall health and well-being and plays a critical role in quality of life (1). However, immigrants, refugees, asylum seekers, and non-permanent residents, in Canada and the United States, continue to experience substantial inequities in access to dental care and oral health outcomes (2). These inequities are a result of systemic, cultural, and socio-economic barriers that have a disproportionate impact on vulnerable populations, including newcomers (1–3). These patterns are often shaped by broader social determinants of health that influence access to resources, health literacy, and healthcare utilization across migrant populations (4).

In this review, key concepts related to oral health equity are used in a complementary but conceptually distinct manner. Disparities refer to observable differences in oral health outcomes or service utilization across population groups. Inequities represent those differences that are considered avoidable, unfair, and systematically associated with social disadvantage (4). Access to care is understood as one of the mechanisms through which such inequities manifest, encompassing the ability of individuals and populations to obtain appropriate dental services when needed. Equity-promoting strategies refer to policies, programs, or interventions designed to reduce these unjust differences and improve the distribution of oral health resources and outcomes across populations (4, 5).

Given the sizeable and recent migration trends in Canada and the United States, there is an urgent need to consider oral health inequities among newcomers, as both countries have seen increases in both the diversity and size of their migrant populations (1, 6, 7). As these disparities continue, many newcomers face unique circumstances, such as language and cultural barriers, lack of familiarity with the local health system, and limited eligibility for public dental programmes (1–3). Recent reports estimate an increasing proportion of immigrants, refugees, and non-permanent residents, who have migrated to high-income countries, representing a substantial portion of the Canadian and American populations, and reinforce the importance of oral health equity research (6, 7).

Although individual studies and community-based programs have addressed some barriers to oral health equity, there remains a lack of comprehensive synthesis of the evidence on interventions, strategies, barriers, and facilitators related to oral health equity among immigrant populations in Canada and the United States (2, 3). The existing literature often addresses non-immigrant, global, paediatric, or specific ethnic subpopulations, leaving a gap in the literature regarding broader policy environments and the inequitable state of oral health systems in Canada and the United States.

A scoping review is particularly appropriate for this topic, as it allows for the systematic mapping of a diverse and possibly heterogeneous body of evidence; identification of research gaps; and examination of a wide variety of interventions, barriers, and facilitators (8). This methodological approach also enables policymakers, practitioners, and researchers to understand the complex landscape of oral health equity for immigrants and to develop evidence-based, context-appropriate responses.

A preliminary search of MEDLINE, the Cochrane Database of Systematic Reviews, and JBI Evidence Synthesis was conducted. To our knowledge, no scoping or systematic reviews have synthesized the available evidence on interventions, strategies, barriers, and facilitators related to oral health equity among immigrant populations in Canada and the United States. Therefore, this protocol proposes a scoping review to consolidate existing evidence on interventions, strategies, barriers, and facilitators related to oral health equity for immigrant populations in Canada and the United States.

The objective of this scoping review is to systematically map interventions, strategies, barriers, and facilitators related to promoting oral health equity for immigrant populations, including newcomers, refugees, asylum seekers, and non-permanent residents, in Canada and the United States.

## 2 Review question

### 2.1 Main question

What is the extent and nature of the evidence on interventions, strategies, barriers, and facilitators related to oral health equity for immigrants, including newcomers, refugees, asylum seekers, and non-permanent residents, in Canada and the United States?

### 2.2 Sub-questions

1. What types of interventions or strategies have been reported to enhance oral health equity for diverse immigrant populations?
2. How have these approaches been implemented, and what outcomes have been described for immigrants, refugees, and non-permanent residents?
3. What barriers and facilitators to oral health equity for these populations have been identified in the existing literature?

## 3 Inclusion criteria

### 3.1 Population

This review will consider studies addressing immigrants, refugees, asylum seekers, and non-permanent residents living in Canada or the United States. These groups include individuals who have migrated for economic, family, or humanitarian reasons, as well as those fleeing persecution or conflict, and temporary residents such as international students and workers.

Eligible studies will include populations across all life stages and demographic groups, from children and adolescents to adults and older adults. Evidence addressing specific subpopulations, such as culturally and linguistically diverse groups or individuals with low income, will also be included to explore how age, socioeconomic status, and culture intersect to influence oral health inequities.

Studies will be considered if they examine the oral health conditions, access to dental services, or experiences of these populations within the healthcare system. Both quantitative and qualitative primary research, as well as policy reports and relevant grey literature, will be included. Studies will be excluded if they do not specifically address oral health or equity for immigrant populations, focus on native or long-term residents, lack empirical or contextual data, or are published in languages other than English.

## 4 Concept

The review will explore the concept of oral health equity, defined as the fair and just distribution of oral health resources, access to care, and outcomes across immigrant populations in Canada and the United States. This definition aligns with broader frameworks of health equity that emphasize the role of social determinants of health in shaping unequal access to healthcare services. The aim is to identify the range of barriers, facilitators, and strategies designed to reduce inequities and promote equitable access to dental services.

Barriers include financial constraints such as treatment costs and lack of insurance coverage; language and cultural differences that hinder communication and care-seeking; and structural barriers, such as the limited integration of dental services within public health systems. Facilitators and interventions include culturally appropriate programs, community- and school-based initiatives, health literacy efforts, and policy-level actions to expand coverage or improve service accessibility.

Studies will be excluded if they solely assess clinical treatment efficacy without addressing equity or access, or if they lack relevance to immigrant oral health in Canada or the United States.

## 5 Context

The review will be conducted within the context of Canada and the United States, two major immigrant-receiving countries with distinct healthcare systems but comparable challenges in addressing equity. Canada, which has one of the highest immigrant shares among developed nations, and the United States, home to the largest number of immigrants globally, both countries have culturally diverse populations and policy environments that influence immigrants’ experiences in accessing oral health care (9, 10).

Cultural and linguistic diversity significantly influences oral health beliefs, behaviours, and communication with providers. Geographic variation, particularly between urban and rural regions, further affects access, with rural areas often facing limited dental infrastructure and workforce shortages (11).

At the system level, differences in healthcare models contribute to persistent disparities. In Canada, dental care is largely excluded from universal healthcare, leaving many immigrants reliant on private insurance or out-of-pocket payments (12). In the United States, access is determined largely by state-level Medicaid policies and private insurance coverage, resulting in substantial variability in eligibility and benefits (13).

This review will also consider population-specific contexts, including the high prevalence of dental caries among immigrant children, the influence of maternal oral health and caregiving responsibilities on women’s access to care, and the compounded barriers experienced by older immigrants due to cost and mobility limitations.

## 6 Types of sources

This scoping review will include quantitative, qualitative, and mixed-methods primary studies addressing oral health equity among immigrant populations in Canada and the United States. Eligible evidence will encompass experimental, quasi-experimental, and observational designs, as well as descriptive studies such as large case series and surveys. Relevant policy documents, government and organizational reports, and other grey literature providing empirical or contextual insights will also be considered. Small case reports and narrative or opinion papers lacking empirical data will be excluded.

## 7 Methods

The proposed scoping review will be conducted in accordance with the JBI methodology for scoping reviews (14, 15) and reported following PRISMA-ScR guidelines (16). This protocol is registered in the Open Science Framework (OSF) (registration link: https://doi.org/10.17605/OSF.IO/PDNEM).

### 7.1 Search strategy

A three-step search strategy will be employed in this review to ensure a comprehensive and systematic identification of relevant studies. An initial limited search of MEDLINE (via PubMed) and Scopus will be conducted to identify key articles on the topic. The text words contained in the titles and abstracts of relevant papers, along with the index terms used to describe them, will be analyzed to develop a complete search strategy.

The finalized strategy will then be applied to the selected databases, including MEDLINE (via PubMed) for biomedical and public health literature, Scopus for multidisciplinary research across health and social sciences, Web of Science for high-impact journals in health, policy, and migration, CINAHL (via EBSCO), and Embase for biomedical and health sciences literature.

Grey literature sources will also be searched to capture unpublished reports and policy documents relevant to oral health equity. These will include OpenGrey, Google Scholar, and selected governmental and institutional websites relevant to oral health and migration policies (e.g., Health Canada, Centers for Disease Control and Prevention, Immigration, Refugees and Citizenship Canada). The first 200 results sorted by relevance will be screened in Google Scholar. In addition, the reference lists of all included studies will be screened to identify further eligible sources of evidence.

Only studies published in English will be included due to the predominance of English-language publications in oral health and public health research in Canada and the United States and the lack of resources available for translation. No restrictions will be applied regarding study design, provided the studies meet the inclusion criteria. The complete preliminary PubMed search strategy, including MeSH terms, keywords, and Boolean operators, is presented in Supplementary Appendix I and will be adapted for Scopus, Web of Science, CINAHL, and Embase according to each database's indexing terms.

### 7.2 Study/source of evidence selection

Retrieved studies will be uploaded into Zotero (Corporation for Digital Scholarship, 2024), where duplicates will be automatically removed. Screening will be performed in two stages by pairs of independent reviewers, following prespecified eligibility criteria.

Stage 1 will involve screening titles and abstracts to identify potentially relevant studies. Title and abstract screening will be conducted using Rayyan (17) (Qatar Computing Research Institute) which allows reviewers to apply eligibility criteria and use highlighting and annotation features to support consistent decisions. A standardized screening form will be developed in Microsoft Excel (Redmond, Washington, USA) to capture study characteristics and eligibility outcomes.

Stage 2 will consist of full-text screening for all studies identified in Stage 1. Screening forms will again be piloted on at least 10 articles to ensure consistency, and revisions will be made as needed. Reasons for exclusion at the full-text stage will be documented systematically. Any disagreements during the screening process will be resolved through discussion or adjudication by a third reviewer. All included sources of evidence will be imported into the JBI System for the Unified Management, Assessment, and Review of Information (JBI SUMARI; JBI, Adelaide, Australia) for data management and extraction. The entire selection process will be reported in accordance with the PRISMA-ScR guidelines, and a flow diagram will be generated to depict the number of records identified, screened, included, and excluded at each stage.

### 7.3 Data extraction

Two independent reviewers will extract data from eligible studies using a standardized data extraction tool developed in JBI SUMARI (13). The extraction form will be piloted before full data charting to ensure consistency and clarity. The following variables will be extracted from each included study:

- Author(s) and year of publication
- Country of study
- Study design (e.g., observational, qualitative, review, policy report)
- Population characteristics (e.g., type of newcomers, age group, gender, socioeconomic status)
- Context (e.g., healthcare setting, community, policy framework)
- Barriers to oral health care
- Facilitators to oral health care
- Interventions/approaches described
- Outcomes related to oral health access or equity
- Key findings and implications

Any disagreements regarding data extraction will be resolved through discussion or consultation with a third reviewer. Authors of included studies may be contacted for clarification or additional information if needed. Consistent with scoping review methodology, no formal critical appraisal of individual studies will be conducted (13). However, methodological characteristics of included studies will be reported to support interpretation of the findings. Any modifications to the data extraction tool during the review process will be documented and reported.

### 7.4 Data analysis and presentation

Extracted data will be analyzed using a combination of descriptive and thematic synthesis. Quantitative data will be summarized using descriptive statistics, including frequencies and distributions of study characteristics, populations, and types of interventions. Qualitative data will undergo thematic content analysis to identify recurring concepts, patterns, and relationships among barriers, facilitators, and interventions related to oral health equity. Coding will be conducted inductively, allowing themes to emerge from the data. Two reviewers will independently code the extracted qualitative information, and discrepancies will be resolved through discussion or consultation with a third reviewer when necessary. The analytic process will be iterative, with codes and themes refined throughout the review.

Emerging themes will be organized according to the main domains of the review, including barriers, facilitators, and interventions influencing access to oral health services among immigrant populations. To support interpretability, findings will also be mapped across ecological levels, including individual-level factors, community-based initiatives, and structural or policy-related determinants influencing oral health equity.

Results will be presented in summary tables and figures organized according to the review questions and domains. A narrative synthesis will accompany the tabulated findings to contextualize and interpret the evidence within the broader framework of oral health equity for immigrant populations in Canada and the United States.

## Data Availability

The original contributions presented in the study are included in the article/Supplementary Material, further inquiries can be directed to the corresponding author.
