# Supplementary material for: Strategies to promote oral health equity for immigrant populations in Canada and the USA: a scoping review protocol
Source: Front Oral Health. 2026 Apr 10;7:1804288. doi: 10.3389/froh.2026.1804288 (PMC13106570; doi:10.3389/froh.2026.1804288)
Supplement: Supplementary file 1 [file Supplementaryfile1.docx]

# **Supplementary Material**

## **Appendix**

**Appendix I: Full PubMed Search Strategy (MEDLINE via PubMed)**

| **Concept** | **MeSH terms** | **Keywords (free-text)** | **Boolean/Notes** |
| --- | --- | --- | --- |
| **Population (newcomers)** | "Emigrants and Immigrants"[MeSH]; "Refugees"[MeSH]; "Asylum Seekers"[MeSH] | immigrant*, refugee*, “asylum seeker*”, newcomer*, “non-permanent resident*” | Combine with OR |
| **Oral health** | "Oral Health"[MeSH]; "Dental Care"[MeSH]; "Oral Hygiene"[MeSH] | “oral health”, “dental care”, “oral hygiene”, dentistry | Combine with OR |
| **Equity/Access** | "Health Equity"[MeSH]; "Healthcare Disparities"[MeSH] | equity, disparities, barrier*, facilitator*, intervention*, access | Combine with OR |
| **Geography** | "Canada"[MeSH]; "United States"[MeSH] | Canada, “united states” | Combine with OR |

**Appendix II: Final Search String (PubMed)**

(("Emigrants and Immigrants"[MeSH] OR "Refugees"[MeSH] OR "Asylum Seekers"[MeSH]

OR immigrant*[tiab] OR refugee*[tiab] OR "asylum seeker*"[tiab] OR newcomer*[tiab]

OR "non-permanent resident*"[tiab])

AND

("Oral Health"[MeSH] OR "Dental Care"[MeSH] OR "Oral Hygiene"[MeSH]

OR "oral health"[tiab] OR "dental care"[tiab] OR "oral hygiene"[tiab] OR dentistry[tiab])

AND

("Health Equity"[MeSH] OR "Healthcare Disparities"[MeSH]

OR equity[tiab] OR disparities[tiab] OR barrier*[tiab] OR facilitator*[tiab]

OR intervention*[tiab] OR access[tiab])

AND

("Canada"[MeSH] OR "United States"[MeSH]

Canada[tiab] OR "united states"[tiab]))

Filters: English[lang]
